# Challenges and Prospects for the Conservation of Crop Genetic Resources in Field Genebanks, in In Vitro Collections and/or in Liquid Nitrogen

**DOI:** 10.3390/plants9121634

**Published:** 2020-11-24

**Authors:** Bart Panis, Manuela Nagel, Ines Van den houwe

**Affiliations:** 1 Alliance of Bioversity International and CIAT, c/o KU Leuven, Willem de Croylaan 42, P.O. Box 2455, 3001 Leuven, Belgium; i.vandenhouwe@cgiar.org; 2 Department of Biosystems, KU Leuven, Willem de Croylaan 42, 3001 Leuven, Belgium; 3 Leibniz Institute of Plant Genetics and Crop Plant Research (IPK), OT Gatersleben, Corrensstrasse 3, D-06466 Seeland, Germany; nagel@ipk-gatersleben.de

**Keywords:** clonal crops, collection management, cryobiotechnology, cryopreservation, field collections, field maintenance, germplasm storage, in vitro conservation, recalcitrant seeds

## Abstract

The conservation of crop genetic resources, including their wild relatives, is of utmost importance for the future of mankind. Most crops produce orthodox seeds and can, therefore, be stored in seed genebanks. However, this is not an option for crops and species that produce recalcitrant (non-storable) seeds such as cacao, coffee and avocado, for crops that do not produce seeds at all; therefore, they are inevitably vegetatively propagated such as bananas, or crops that are predominantly clonally propagated as their seeds are not true to type, such as potato, cassava and many fruit trees. Field, in vitro and cryopreserved collections provide an alternative in such cases. In this paper, an overview is given on how to manage and setup a field, in vitro and cryopreserved collections, as well as advantages and associated problems taking into account the practical, financial and safety issues in the long-term. In addition, the need for identification of unique accessions and elimination of duplicates is discussed. The different conservation methods are illustrated with practical examples and experiences from national and international genebanks. Finally, the importance of establishing safe and long-term conservation methods and associated backup possibilities is highlighted in the frame of the global COVID-19 pandemic.

## 1. Introduction

In the course of crop domestication, many plants have been selected for quantity and/or quality of their seed, while some have been cultivated for their roots, tubers, fruits, stems and leaves. Plant genetic resources for food and agriculture (PGRFA) are of strategic importance to ensure sustainable crop production [1], nutritious food and food security for humans and to enhance economic prosperity of the present and future generations. They comprise the sum of genes, gene combinations or genotypes which serve as a reservoir for direct use in food production systems and for breeding new varieties [2].

Since the beginning of agriculture, selection of plants and seeds for sowing, growing, harvest and storage gave rise to locally adapted varieties, so-called “landraces”, that reveal specific variations of morphological and yield characteristics and quality traits. In the mid-19th century, the rediscovery of Gregor Mendel’s work and the introduction of breeding schemes resulted in the development of high-yielding and more stress-tolerant varieties leading to higher crop yields. This laid the foundation for the green revolution taking place in the middle of the last century bringing about increased agricultural production to feed the exponentially growing world population. However, the expansion of industrial mono-cropping with the replacement of landraces by modern breeding varieties has caused the loss of 75% of plant genetic diversity, with more than 90% of crop varieties having disappeared from farmers’ fields [3]. In this era of global environmental problems, climate change, and booming population growth, it is of paramount importance that the remaining crop genetic resources are kept available to sustain the agricultural production systems, to feed the world population a healthy diet and to tackle future demanding challenges [4].

Since the 16th century, botanical gardens have collected and preserved a variation of more than 80,000 plant species in about 3400 gardens all over the world [5]. They primarily have an interest in conserving the widest possible plant diversity and crop wild relatives that can be an important source material for breeders. PGRFA are conserved ex situ in specialized repositories, often termed genebanks that have been established since the mid-20th century. In contrast to most botanical gardens, genebanks focus on both intra- and inter-specific crop diversity. There are more than 17,000 national, regional and international institutes and organizations dealing with the conservation and sustainable use of PGRFA [5]. Currently, 711 gene banks and 16 international/regional centers in 90 countries retain more than 5.4 million accessions from over 7051 genera, mainly focusing their conservation efforts on crop species, including landraces and crop wild relatives, breeding materials and cultivars [6].

Most of the major food crops produce orthodox seeds that tolerate intense dehydration and low temperatures, thus seed storage under dry and cool conditions is naturally the most widely adopted method for long-term ex situ conservation at relatively low costs. About 45% of the accessions stored as seeds are cereals, i.e., wheat (*Triticum aestivum*), triticale (*Triticum secale*), rice (*Oryza sativa*), oat (*Avena sativa*), rye (Secale cereale), barley (*Hordeum vulgare*), maize (*Zea mays*) and sorghum (*Sorghum bicolor*), followed by food legumes (15%), forages (9%) and vegetables (7%) [5].

In contrast, a large number of food crops are not storable through seeds and thus need different conservation approaches [7]. This category of plants consists of important species that produce desiccation sensitive, recalcitrant or intermediate seeds, such as coconut (*Cocos nucifera*), cacao (*Theobroma cacao*), avocado (*Persea americana*) and citrus (*Citrus* spp.) and species that are seedless such as edible banana (*Musa* spp.) and garlic (*Allium sativum*). Species including yucca (*Yucca* sp.) and bamboo (*Bambuseae* sp.) that have long life cycles and take years or decades to reproduce also fall into this category. Other species that produce orthodox seeds but require the conservation of particular gene combinations or genotypes, such as root and tuber crops, notably potato (*Solanum tuberosum*), cassava (*Manihot esculenta*), yam (*Dioscorea* spp.), taro (*Colocasia esculenta*) and several fruit and nut trees are also included. These crops are propagated vegetatively, and each genotype needs to be maintained as a clone.

**Figure 1:**
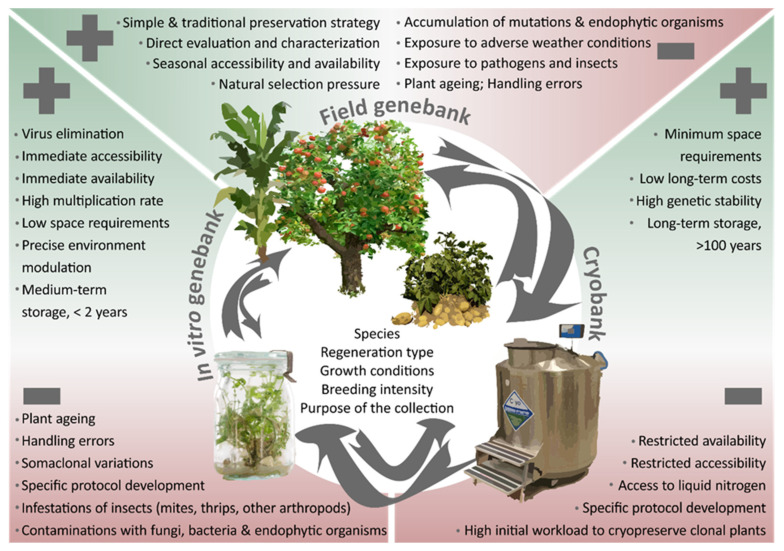
Pros and cons of storing crop genetic resources in field genebanks, in vitro and through cryopreservation. Arrows indicate the potential source material and target approach to maintain plant genetic resources for food and agriculture (PGRFA) and can be specific for each plant species.

Two main ex situ conservation approaches can be distinguished for these crops: the conservation of plants in field genebanks and the maintenance of propagules in tissue culture, either (i) as active growing cultures in short- and medium-term storage (i.e., in vitro storage), or (ii) in frozen state at ultra-low temperature in liquid nitrogen for long-term storage (cryopreservation). These approaches, the challenges entailed, and prospects offered to secure crop diversity ex situ will be discussed in detail in this chapter and are depicted in Figure 1.

## 2. Field Genebanks

Clonally propagated crops and their wild relatives belong to some 34 plant families, including herbs, shrubs, trees and vines [8]. Agronomically important genotypes that were selected over centuries for their specific properties can only be conserved in a vegetative mode. Consequently, the simplest and most traditional way to establish a collection is to gather specific genotypes from farmers’ fields, gardens or in the wild and then grow them in the field genebanks where they continue to grow when maintained appropriately. Even under the highest standards of management, germplasm maintained in the field can deteriorate due to a wide variety of climate conditions, ageing of the plants, diseases and pests, hence the need for timely regeneration. For example, depending on the rootstock and orchard conditions, apple (*Malus* sp.) trees may need to be repropagated periodically after 25–50 years [9].

**Figure 2:**
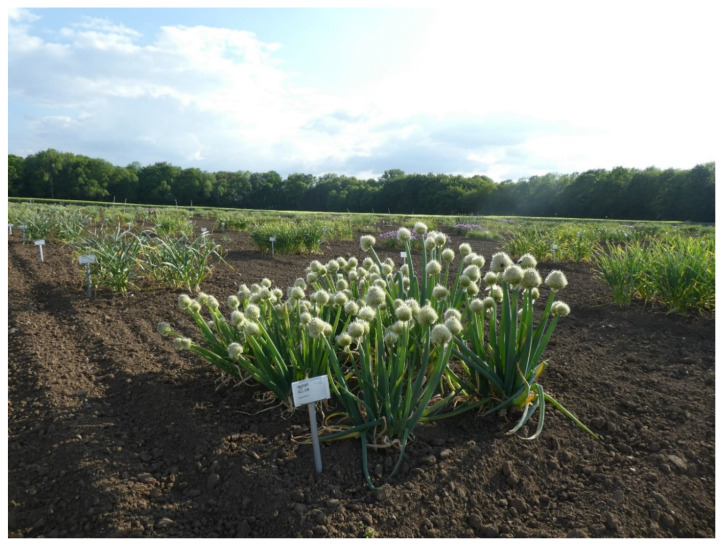
*Allium* field collection at IPK, Germany.

Some of the largest collections were established at the beginning of the 20th century and are based on the efforts of passionate geneticists and plant explorers such as Nikolai I. Vavilov, Frank N. Meyer [10] and Hans Stubbe among others [11]. In the 1970s, the International Board for Plant Genetic Resources (IBPGR) promoted and sponsored numerous collection missions. To preserve clonal material, 23 field genebanks of nine major crops were established worldwide [12]. As such, approximately 400,000 accessions are currently held in field genebanks of international, regional and national authorities. A recent study commissioned by the Alliance of Bioversity International and the International Center for Tropical Agriculture (CIAT), the International Potato Center (CIP) and the Global Crop Diversity Trust [13] showed that of the 20 institutions surveyed, the vast majority of the clonal plant material is kept in the field. Worldwide, major genebanks maintain potato (98,285 accessions), apple (59,922), cassava (36,529), citrus (36,410), sweet potato (*Ipomoea batatas*, 35,478), coffee (*Coffea* spp., 30,483) and cacao (23,107). The largest field genebanks are located in the USA (potato, sweet potato and apple), Japan (apple, citrus and sweet potato), Russia (potato and apple) and Brazil (citrus and coffee). In addition, there are numerous smaller collections, such as for grape vine (*Vitis vinifera* L.), garlic (Figure 2, see Box 1), Jerusalem artichoke (*Helianthus tuberosus* L.), and Andean root crops, which are of high value as luxury foods and condiments or are of regional or religious significance.

### 2.1. Management of Field Genebanks

The specific needs of a crop with respect to growth requirements and its multiplication cycle ultimately determine the field conditions and the structural design of the field genebanks. Propagules of some annual crops, such as potato, shallot or yams, are cultivated by up to 10 plants in the field and require good agricultural practices including crop rotation. After harvest, these propagules must be kept under suitable storage conditions until the next growing season [14]. By contrast, woody crops, such as apple, pear, coffee and grapevine are grown at the same locations for many years, often with two plants per accession. Some of these crops require budding or grafting to rootstock resistant to root nematodes, layering or rooting stem cuttings. For example, the US Department of Agriculture—Agricultural Research Service (USDA-ARS) National Plant Germplasm System (NPGS) maintains 5004 apple accessions in the field and 1603 *M. × domestica* seed accessions in Geneva, NY, USA. Due to the large number, about 3100 field accessions are only represented by a single tree [15]. By comparison, the JKI Dresden-Pillnitz, but also universities, governmental institutes, communes, non-governmental organizations and private individuals are responsible for the maintenance of fruit collections in Germany. These collections include landraces that partly date back to the 12th century and some of these are at risk. Therefore, the German Fruit Genebank was launched in 2007 and consists of six fruit-specific networks (apple, cherry and plum (both *Prunus* spp.), berries (*Rubus* spp.), strawberry (*Fragaria* spp.), pear). For apple, an expert group selected 743 unique accessions that were duplicated and are represented by two trees each in at least two sites [16]. Although, establishing networks requires additional efforts, the responsibility for maintaining such valuable resources is shared and the partners mutually benefit from the concerted expertise and the security status of the collections.

Depending on the collection strategy and the breeding programs, both annual and perennial crops can vary in proportions of landraces, breeding lines and cultivars and are used as different types of donors for plant breeding. CIP, for example, maintains the largest collection of 4487 potatoes landraces at Huancayo, Peru at a 3200 m elevation. [17]. To increase the productivity and, hence, farmer’s incomes in Africa, Asia and Latin America, CIP and partners developed training guides for the positive selection of propagules of landraces that have no visible symptoms of diseases or abiotic stress [18]. By comparison, at IPK (Leibniz Institute of Plant Genetics and Crop Plant Research), in addition to 3300 landraces, also 2900 wild potato accessions are maintained as orthodox seeds. As some potato species are self-incompatible, seeds are propagated as a population in the greenhouse and are screened for resistances, to late blight (*Phytophthora infestans*) on tubers or pale potato cyst nematodes (*Globodera pallida*) [19]. Selected donors are further used to develop introgression lines that can be later used by the breeding industry. In other words, breeding intensity and objectives determine the composition and have a strong impact on the management system of the collections.

Box 1 **Field collection of Allium genetic resources at IPK, Germany.**

The IPK maintains one of the largest *Allium* collections worldwide and comprises the Taxonomic Reference Collection of 1300 accessions and 287 species and the *Allium* Crop Collection covering 1400 accessions of 76 species (Figure 2). About 2100 accessions are permanently maintained in the field because of (i) their inability to form seeds, i.e., in garlic and shallots (*Allium cepa* var. *aggregatum*), (ii) the presence of heterozygous seeds, i.e., in onion (*Allium cepa* var. *cepa*), or (iii) the traditional breeding strategy as clonal varieties as in the case of some ornamental *Allium* hybrids [20].

*Allium* accessions, except shallot and onion, are maintained as a permanent crop at the same site and 3 to 6 plants are planted within a 2 m^2^ plot. These accessions require permanent management including regular identification, weeding, phytosanitary treatments and seed harvest to avoid the establishment of different accessions or hybrids within the same plots. The soil quality and the continental climate in Central Germany, including mild winters, reduce the risk of pest and diseases and provide optimum growth conditions for a longer term. However, each 5 or 6 years, the Allium gardens are replanted to overcome the problem of soil exhaustion [21].

Special attention must be given to 425 garlic accessions and 82 shallot accessions that are partially replanted every year. In autumn, to avoid infections of nematodes and wireworms, cloves are treated with insecticides and planted in the field. At the end of July of the following year, accessions develop cloves (lateral bulbs) and in some cases bolt and develop bulbils which dry up. These can be used for germplasm distribution. For introduction into tissue culture or cryopreservation, bulbs and bulbils require a further after-ripening period of up to 2 months until the physiological dormancy [22] is broken. During this period, storage of the bulbs and, in the case of garlic also the bulbils, is conducted at between 4 and 10 °C [23].

Various strategies have been developed to support the field genetic resources of garlic and shallot. About 30 years ago, in vitro slow-growth maintenance at a temperature between 2 and 10 °C was initiated for about 700 accessions. As such, cultures can be kept for up to 12 months without subculturing. However, after many subculture cycles, the accumulation of microbes delimitates the in vitro storage. Therefore, the number of in vitro accessions was reduced to about 25 accessions. Nowadays, the introduction of garlic and shallot accessions into in vitro conditions is only carried out as preparatory step for cryopreservation. Since the PVS3 vitrification approach has been successfully developed for garlic and shallot [24], more than 210 accessions (Table 1) were successfully cryopreserved. To protect further the allelic diversity of garlic, the Research Institute of Crop Production (CRI) in the Czech Republic, the Research Institute of Horticulture (RIH) in Poland and the IPK established a European Core Collection within the frame of “A European Genebank Integrated System” (AEGIS) [25].

### 2.2. Advantage of Field Collections: Characterization and Evaluation

The propagation of clonal plants in the field is the conventional method for the preservation of genetic material and traditional knowledge of the farming system [26]. In areas where plants are historically grown, the cultivation can be carried out by local farmers. The sites are usually well-established with pest control and forecasting models used [27]. Their exposure to natural conditions allows a limited selection according to environmental conditions and a competition between plant propagules [8]. A tremendous benefit of holding field collections is that images and voucher specimens can be immediately assessed and made available online [27,28,29].

Descriptors have been elaborated for a wide range of crop species [27,29,30]. The descriptors and the list of Multi-Crop Passport Descriptors provide an international format that facilitates comparisons among and within collections [14]. The data often follow FAIR—findable, accessible, interoperable and reusable—principles [29] and well-characterized material can be further distributed and evaluated and made available to breeding programs. At IPK in 2019, of 3300 maintained potato cultivars, about 450 accessions were propagated in the field and of these 1027 sub-samples were distributed. A main advantage is that the material is immediately available for evaluation and distribution. For example, at a field-based germplasm collection in New Zealand genomic DNA was extracted and screened for the presence of TG689 and 57R haplotypes linked to the *H1* gene. These haplotypes act as potential predictors for the resistance against a pathotype of potato cyst nematode (*Globodera rostochiensis*), an economically important pest [31]. Similarly, banana germplasm was screened for the resistance against the *Fusarium oxysporum* f. sp. *cubense* tropical race 4 (*Foc TR4*) that has seriously threatened global banana production. From 129 evaluated accessions, 10 were highly resistant to the virus [32]. The readily available data and material can thus have a strong impact on the material selected for breeding.

### 2.3. Problems Associated with Field Collections

In field collections, plants are exposed to a natural environment, which can include unfavorable climatic conditions, such as drought, heat or frost, similar to crop production fields. Additionally, pests and diseases can threaten the material, especially the less adapted or susceptible accessions. One of the important apple diseases, fire blight, caused by the bacterium *Erwinia amylovora,* can severely damage or even eradicate susceptible apple accession [33,34]. In 2013, the *Allium* field collection at IPK was challenged with a serious infestation of larval stage click beetles (*Elateridae*), also known as wireworms. Of 2150 accessions, 52 accessions were completely lost and 73 could be recovered by replanting and duplication. Furthermore, depending on the plant species and their life cycles, clonal collections must be rejuvenated periodically. The Tropical Agricultural Research and Higher Education Center (CATIE) in Costa Rica hosts one of the largest collections of *Coffea arabica* L. and maintains plants that were planted in the 1970s. In the year 2000, a project to rejuvenate the collection was initiated and is still ongoing [14,35]. Compared to reproduction by seeds, vegetatively propagated populations are also susceptible to the accumulation of viruses, bacteria and fungi that might be similar to Muller’s ratchet [8]. As a consequence, the maintenance of field collections requires substantial efforts in terms of agricultural measures such as manual selection, evaluation, mechanical weed control, rejuvenation and specific disease control. Therefore, the number of replicates is often limited to between 5 and 10 for cassava, 10 and 12 for sweet potato, 2 and 9 for garlic, 1 and 3 for trees and shrubs and 3 and 20 for bananas [14]. However, spatially separated replicate plants (i.e., trees and vines) can aid in minimizing loss of accessions by pests or mismanagement. However, even provided everything is handled with the utmost care and state-of-the-art technologies, human errors can easily happen and accessions can be mixed up. A genotyping study of 250 accessions of the CIP potato collection showed that only about 80% of the collection was still comparable with voucher mother plants which arrived at CIP about 30 years before [36]. At Arabidopsis stock centers, it was estimated that about 3 to 14% of the materials are misidentified with most errors caused by incorrect labeling [37]. There is also the possibility that genetic lines segregate and spontaneous mutations occur. Especially under environmental stress, whole-genome and whole-methylome sequencing revealed that the Arabidopsis lineages accumulate 100% more genetic mutations and epigenetic modifications under stress compared to non-stress conditions [38]. This underlines the importance of cautious evaluation, involving trained staff, and the application of complementary preservation methods such as in vitro storage and cryopreservation.

## 3. In Vitro Collections

In vitro culture (or tissue culture) of plants is a biotechnological technique in which plant parts are isolated from in vivo plants, disinfected to free the explants from bacteria and fungi and transferred onto well-defined and sterile tissue culture media that provides the plant tissue with the necessary nutrients for growth and multiplication. In a relatively small space, the environment can be controlled precisely and plant growth can be easily observed and manipulated. In vitro approaches are commonly used for large-scale micro-propagation, reproduction purposes including embryo rescue, ploidy manipulations, protoplast fusions and somatic embryogenesis and are appropriate tools for short- and mid-term storage of plant genetic resources. Although the feasibility of using in vitro culture methods for plant genetic resources conservation was already known in the 1970s, it was only in the 1980s that the International Board for Plant Genetic Resources (IBPGR) established a working group of specialists to investigate the critical aspects of in vitro plant conservation [39,40]. Since then, in vitro collections have been setup for many vegetatively propagated crops. Additionally, Genebank Standards for PGRFA maintained in vitro were developed [1], forming the benchmark for establishing standard operating procedures and quality management systems to ensure effective, safe and efficient conservation of these genetic resources. Nowadays, in vitro collections for PGRFA comprise potato (9700 accessions), cassava (8700), sweet potato (6400), yam (3200), banana (2000) and taro (1200) [40,41,42]. The largest collections are maintained by international organizations such as Bioversity International, the International Center for Tropical Agriculture (CIAT), CIP, the International Institute of Tropical Agriculture (IITA) and in national institutes such as Brazilian Agricultural Research Corporation (EMBRAPA) in Brazil, CRI in Czech Republic, the IPK in Germany and the USDA-NPGS in the USA [13,42].

### 3.1. Setting Up In Vitro Collections

At tissue culture facilities, plant tissues are maintained in specific and aseptic growth conditions. To culture explants in vitro, typically tissue culture media are used that contain water, macro and micro nutrients (salts), a gelling agent, plant growth regulators (plant hormones) and sugars. The supplement of sugars as source of energy and building blocks is essential because, unlike in vivo plants, in vitro plants lack the ability to photosynthesize effectively. Explants are kept in a culture room with controlled temperature and light regimes. Since tissue culture material continuously grows and undergoes ageing, plants regularly need to be trimmed, divided in separate propagules and transferred to new culture media. Explant survival and vitality of in vitro plants depend on media composition, temperature and light intensity and further factors such as light spectrum, vessel type and size, number of explants and gas exchange rate [43,44,45], all of which can affect the growth of the plants.

**Figure 3:**
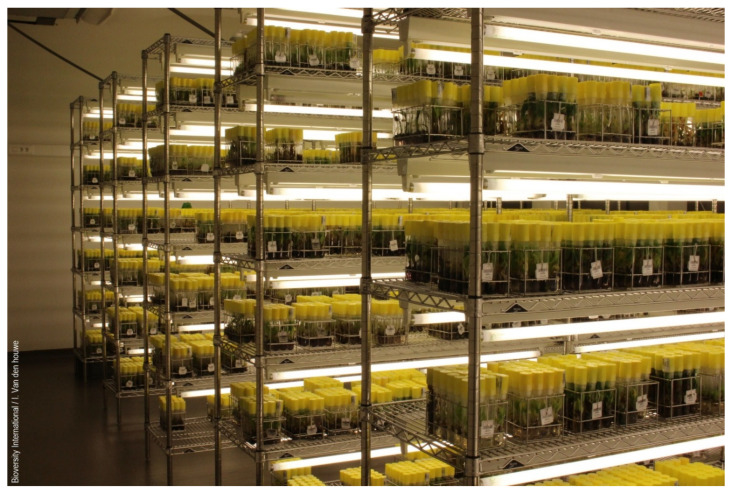
Banana in vitro collection at the International Transit Centre (ITC), Belgium.

The goals of applying slow-growth conditions in plant collections is to reduce frequency of subculture events that are labor (and thus cost) intensive and to minimize the risk of loss of accessions due to handling errors and genetic instability induced by the tissue culture environment. Under optimal growth conditions, subculture frequencies range from one to three months, whereas at slow growth conditions, the subculture period can be from one to two years. Three so-called Medium Term Storage (MTS) approaches can be distinguished: physical growth limitation, chemical growth limitation and nutrient limitation [40]; all aimed to reduce the metabolic activity of the plantlets. Physical growth limitation is often achieved by lowering temperature and often combined by applying low light intensities. Cold-tolerant species such as garlic, potato and most *Mentha* species can be kept viable at temperatures from 0 to 5 °C and 2 to 4 μmol m^−2^ s^−1^ for up to 24 months [46], whereas cold sensitive species such as many tropical plants should be stored at relatively high temperatures. Examples are *Musa* [47] (Figure 3, see Box 2) and sweet potato [48] both requiring a temperature of 15 °C or higher; for pineapple this is 21 °C [49]. Such higher storage temperatures result in shorter sub-culture intervals (six to twelve months). Nutritional growth limitation decreases the supply of carbon and inorganic nutrients whereas chemical growth limitation involves the application of osmotically active agents such as mannitol, sorbitol, polyethylene glycol (PEG) or growth retardants such as abscisic acid (ABA) and hydrazides. In potato, for example, extended periods in MTS can be achieved by different protocols which either apply Murashige and Skoog (MS) medium, sucrose and the growth retardant ancymidol at 6 °C resulting in storage periods of 12 months [50]; MS and mannitol at 6 °C for storage periods of 16 months [51] or MS, sucrose, mannitol at 6 °C for storage periods of 30 months [52]. This shows that often combinations and variations of different slow-growth approaches need to be adapted for different species. An overview of growth limiting protocols is summarized by Chauhan, Singh and Quraishi [53].

### 3.2. Advantages of In Vitro Collection

An important benefit of in vitro collections, compared to field collections, is that the plant material is free of most pests and diseases. An exception is in the case of viruses, which can easily be transmitted through tissue culture, often symptomless. As international quarantine restrictions are very stringent for some species, a prerequisite for germplasm to be internationally distributed is that the sample must be healthy and free of harmful pathogens. Several in vitro tissue culture techniques, such as thermotherapy, chemotherapy and meristem tip culture, can be applied to eradicate the viruses [42]. In addition, other eradication methods such as electrotherapy [54] and cryotherapy [55] have been effective in several crops including grape, potato, sweet potato and banana. Cryotherapy using vitrification proved to eliminate effectively Cucumber Mosaic Virus (CMV) and Banana Streak Virus (BSV) from in vitro meristematic tissues of the dessert banana cv. Williams (AAA, Cavendish subgroup) with 30% and 90% of the regenerated plants being CMV- and BSV-free, respectively [56]. Often, combinations of eradication techniques are used to increase their effectiveness.

In contrast to field gene banks, plant materials kept at MTS conditions, also called in vitro “active” collections can be delivered on a year-round basis. For most crops, the availability of samples from a field gene bank is bound to the development stage of the plant and season. In garlic, for example, cloves and bulbils follow a seasonal development and are only available and highly viable between July and February in the northern hemisphere. Moreover, the supply of material from the field can be limited because of the often poor plant propagation rates. For instance, banana has an annual field multiplication rate through suckering as low as five to 20 suckers per year depending on the clone, age of the plant and climatic and culture conditions [57,58]. Furthermore, the international and inter-regional movement of propagules from the field such as suckers of banana, corm pieces or small corms of cocoyam or taro, tubers of oca, ulluco or other plant parts such as wood cuttings of apple or blueberry, involves the risk of transmission of harmful pest and diseases. Strict quarantine measure must thus be taken. For crops with insect- or mite-transmitted viruses, it is therefore useful to maintain virus-free stocks in screenhouses although this implies higher cost for making materials available to users. In general, it is recommended that vegetative material is distributed to requesters as tissue cultures, derived from conventional vegetative propagation material, and indexed free from pathogens. Moreover, tissue culture using multiplication-inducing growth regulators, often cytokinins, allows rapid and mass propagation of plants. Hence, hundreds of propagules can be produced within a few months.

### 3.3. Problems Associated with In Vitro Collections

The artificial tissue culture environment can be stressful for plant cells and thus poses a challenge when plant germplasm needs to be maintained for extended periods of time. This can result in so-called somaclonal variations that are changes in the DNA sequences that are not derived from recombination [59]. It can have epigenetic origin which reflects the adaptation process of cells to a different environment [60]. Different factors have an effect on the genetic mutation rate, among them exposure of the chromosomal DNA to different chemicals present in the culture medium and the survival of the variants in a non-selective tissue culture environment. Although the mutagenic activity of plant growth regulators is debated, it is generally accepted that stimulated rapid disorganised growth induces somaclonal variation [59]. This has been shown for thiadiazuron (TDZ) in bananas [61] and the synthetic auxin 2,4-dichlorophenoxyacetic acid (2,4-D) in *Curcuma* aromatic plants [62]. Bairu, et al. [63] observed that, with increasing subculture events, the frequency of somaclonal variation of micropropagated bananas increased, sometimes as high as 72%. However, in potato, random DNA methylation changes occurred only in individual samples at a very low frequency for 3 of 469 markers [64]. Furthermore, no differences in genetic and epigenetic changes have been reported for in vitro propagated pea clones that have been maintained for more than 24 years [65], indicating that somaclonal variation is strongly species-dependent. A general principle is that, the more disorganized the cultured tissues, the higher chance of mutations [59,66]. Therefore, for slow-growth storage, organized tissue systems, such as shoot cultures, are preferred over non organized tissues like callus and suspension cells and the in vitro storage is preferably conducted on hormone-free culture media, as has been used for *Mentha* species [67].

Other constraints in tissue culture collections are the occurrence of cellular ageing and senescence during prolonged cultivation. In eight-year-old peach palm (*Bactris gasipaes*) cultures, processes of plant cell death and senescence were visible through nuclear condensation, cell degradation and the development of large intercellular spaces [68]. The effect of cellular ageing or senescence may appear in parallel with slow growing endophytic microbes that can accumulate over time. Bacteria or fungi are known to colonize almost all healthy plant tissues without necessarily damaging the host or eliciting any defense responses [69]. For example, the in vitro collection of *Allium* species maintained at slow-growth conditions suffers from the presence of endophytes and the storage duration of individual clones is considerably shortened [70]. At the banana collections at the Bioversity Genebank, the tissue cultures are regularly tested for the presence of slow growing (endophytic) microorganisms using a broad spectrum bacterial growth medium. When this method was first applied, the presence of cryptic contaminants in 5% of the stored germplasm was revealed [71]. Although some endopyhtic strains are known to show plant growth promoting behavior such as PsJN in potato [72], the majority is detrimental for in vitro cultures in case they accumulate.

Aside from the challenges that are related to intrinsic processes linked to in vitro culture, external factors can also result in a complete loss of cultures, such as malfunctions in air-conditioning and lighting systems. Moreover, human errors such as accidental microbial contaminations, physical mixing of accession samples, but also documentation errors, e.g., mislabeling, or misidentification, are even more likely to cause serious problems in the operation of an in vitro genebank [73]. Furthermore, mites, thrips and other small arthropods can cause extensive fungal contaminations in tissue cultures and are difficult to eradicate [14]. Therefore, quality management systems including barcode labelling, cleaning management and regular monitoring of the stored materials should be implemented as standard procedures at slow-growth storage facilities [1]. An extra safety measure is duplicating the collection, either in vitro or in cryopreservation, and preferably in another distant location to ensure that the duplicate is properly secured. For example, the *Mentha* collection at IPK consists of two subsets that are maintained in different culture rooms one kept at 2 °C and one at 10 °C. The CIAT genebank in Colombia sends its duplicates of its cassava in vitro clones off site to CIP, Peru where they are maintained at 23 °C and under a controlled photoperiod. For *Musa* at the Bioveristy genebank, maintaining 70% of its in vitro clones in a cryopreserved base collection, a cryopreserved sample is safely duplicated in IRD France.

Box 2 **Collection of bananas at the Bioversity Genebank, Belgium.**

The international banana collection, also known as the Bioversity International Transit Centre (ITC), Belgium, was founded in 1985. It is the largest repository of *Musa* species in the world holding more than 1600 banana accessions sourced from 38 countries. The genebank, located in a non-banana growing country, does not manage a field collection of its own, but has close links with national and regional field collections around the world, serving as a back-up repository for their accessions. The ITC conserves the widest diversity of cultivated sweet and starchy bananas (75%) belonging to 17 genome groups and 52 subgroups, a representation of the wild genepool with specimens of 34 species (16%), and a range of high yielding and disease-resistant advanced varieties (9%). As bananas are vegetatively propagated with seed fertility limited to the wild forms, ex situ conservation of banana species in the form of in vitro cultures is the most suitable approach when the material should remain available for regeneration, multiplication and international distribution.

Germplasm in the collection is stored as multiple shoot clusters obtained from individual shoot tips. Since the early days, low temperature, combined with light limitation, has been successfully applied as growth-retarding factors to reduce the frequency of subculturing considerably. In the storage room, the temperature is 16 °C and light intensity 25 µMol/m^2^/s (Figure 3). It was demonstrated that under the conditions applied, storage duration is nearly one year on average for the total diversity maintained. However, large differences in transfer interval, ranging between 3 and 22 months, occur among the different genomic groups and even within the same subgroup [47].

To keep the collection alive and in good condition, the active in vitro collection requires close monitoring not only to assess the viability and need for re-culturing of accessions, but also to keep the collection free from contamination [71] which may interfere with the storage and use of the germplasm. In addition, samples of accessions maintained for more than 10 years in vitro are evaluated in the greenhouse from where rejuvenated (renewed) in vitro stocks are established. To keep the levels of somaclonal variation of the stored germplasm as low as possible, older accessions are also systematically grown in the field for an integrity check and in parallel to the field detection of changes in morphology, cytological and molecular (SSR) characterization is performed [74] to confirm the identity of the accession.

Each accession in the active collection has a number of 20 replicate cultures maintained on one single multiplication-inducing growth medium, ensuring safe storage of the clone and direct availability for distribution and use. Over the years, the gene bank has played a crucial role in the international exchange of banana germplasm. A system for the safe movement of germplasm is in place with substantial efforts going into the testing and sanitizing of the collection from banana viruses [75]. Each day, three to four clean in vitro samples of the stored banana accessions are distributed to users somewhere in the world to underpin research, breeding and development activities. Depending on the purposes and needs of the germplasm user, the required growth stage of the material may differ: if no tissue culture capacity is available, in vitro rooted plants for direct soil acclimation can be distributed whereas users may need shoot cultures in the multiplication phase for in vitro studies or as virus-free stock for further in vitro propagation. In any case, it is recommended that recipients are provided with detailed handling instructions, the composition of the growth medium and specifications of the growth conditions. Since molecular techniques have become increasingly important in biodiversity studies, scientists and breeders have a growing interest in accessing the DNA of the widest range of genetic diversity present in collections. To meet this changing need, the Bioversity banana genebank established a collection of freeze-dried leaf tissues isolated from greenhouse plants stored at −20 °C. Leaf samples of some 850 accessions are readily available to customers, forming a low-cost alternative for DNA-banking and for distribution of living plant material that is time consuming to prepare, requires phytosanitary checks, special packaging, and fast shipping to assure arrival in good conditions.

The first report on successful shoot tip cryopreservation of *Musa* species was published in 1996 [76]. Sucrose precultured meristem clumps belonging to seven different genomic groups were rapidly frozen in liquid nitrogen, from which the shoots were regenerated. However, regeneration rates were low (ranging between 0% and 50%) and variable depending on the cultivar. A new protocol, i.e., droplet vitrification, was therefore needed (see Section 4.1.2) [77] resulting in higher and more cultivar-independent regeneration rates (between 50% and 95%). Since then and thanks to funding provided by the World Bank and the Gatsby Foundation, routine cryopreservation of clean and field characterized banana accessions kept in the in vitro collection was established. Such secure funding sources proved to be important since cryopreservation of *Musa* species is a very labor intensive and costly process, especially because hundreds of tiny meristems need to be excised under the binocular microscope. Using the droplet vitrification protocol, one skilled technical staff member can cryopreserve about 40 to 50 *Musa* accessions per year.

**Figure 4:**
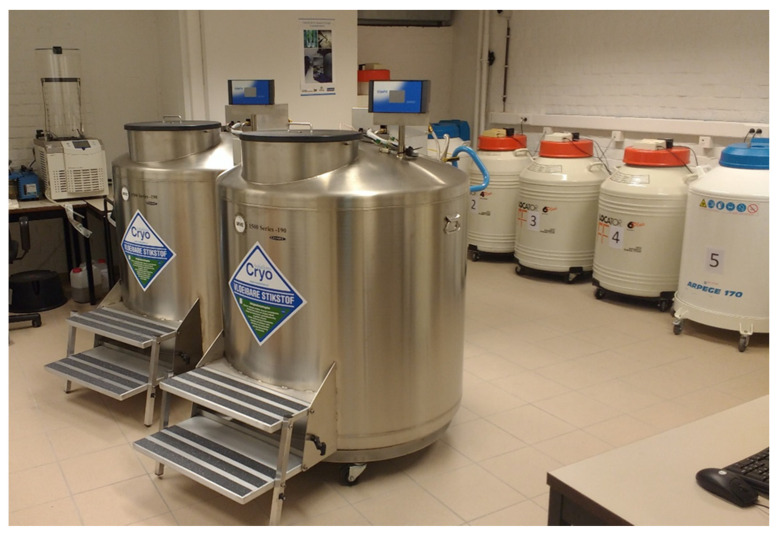
Cryopreservation facilities at the ITC.

The ITC is a successful example of how complementary conservation approaches can be used to increase the security of collections. With samples of 1175 accessions also maintained in liquid nitrogen (Figure 4), the cryopreserved base collection serves as source for replacing materials in case the accession samples in slow growth are lost due to accidental contamination or genetic variations. This cryopreserved collection is “backed up” (duplicated) in Cryotanks held at the Institut de Recherche pour le Development (IRD), Montpellier, France.

## 4. Cryopreserved Collections

Cryopreservation (or storage of biological material at ultra-low temperatures) is the obvious solution to the above-mentioned limitations, since at these conditions metabolic, physical and chemical alterations are unlikely to occur, even after hundreds of years of storage. Usually, cryogenic storage takes place in liquid nitrogen (−196°) or its vapor phase (between −140 and −180 °C) (Figure 4). The main hurdle associated with cryopreservation is the formation of lethal ice crystals. Complete drying plant tissues, thus preventing the formation of ice crystals is not an option since the presence of water is inevitably linked with life. The only way to avoid ice crystal formation of a watery solution is by making use of the physical phase called “vitrification”, i.e., the solidification of a liquid forming an amorphous “or glassy” structure. All cryopreservation procedures developed for biological materials are based on optimizing the chance for vitrification. To attain this, two conditions must be met: (i) application of ultra-rapid cooling rates, limiting the time period that an ice crystal can form before all molecules are immobilized by the ultra-low temperature, and (ii) concentrating the cell solution resulting in relatively more molecules in interference with the organization of water molecules turning into ice crystals.

### 4.1. Setting-Up Cryopreserved Collections

Since the first report by Akira Sakai in 1965 [78] on the survival of plant tissues exposed to liquid nitrogen, a wide variety of plant cryopreservation protocols have been established, among them dormant bud cryopreservation, classical (slow) freezing, encapsulation-dehydration, and a range of vitrifications solution-based protocols (for an overview, see [79]). Currently, dormant bud cryopreservation and droplet vitrification are commonly applied (Table 1).

**Table 1:** Cryopreservation methods used in world’s largest crop genebanks that use cryopreservation for storage of their vegetatively propagated germplasm.

Institute	Country	Crop	Cryopreservation Method	Number of Accessions	Ref
AFOCEL	France	Elm	• Dormant bud freezing	440	[80]
Bioversity International, Leuven	Belgium	Banana	• Droplet vitrification	1100	Panis, personal communication, 2020
Crop Research Institute, Prague	Czech Republic	garlic	• Droplet vitrification	157	[81]
International Center for Tropical Agriculture (CIAT), Cali	Colombia	cassava	• Droplet vitrification • Encapsulation/dehydration	480	[80]
International Institute of Tropical Agriculture (IITA), Ibadan	Nigeria	Yam	• Droplet vitrification	27	[82]
International Potato Center (CIP), Lima	Peru	Potato	• Straw vitrification • Droplet vitrification	3264 (Situation 14 October 2020)	[83]
Julius Kühn-Institut (JKI), Institut für Züchtungsforschung an Obst, Dresden	Germany	Strawberry	• Vitrification	194	[84]
Leibniz Institute of Plant Genetics and Crop Plant Research (IPK), Gatersleben	Germany	Potato,	• Droplet freezing • Droplet vitrification	1818	Nagel, personal communication, 2020
Leibniz Institute of Plant Genetics and Crop Plant Research (IPK), Gatersleben	Germany	Mint	• Droplet vitrification	157	Nagel, personal communication, 2020
Leibniz Institute of Plant Genetics and Crop Plant Research (IPK), Gatersleben	Germany	Garlic and shallot	• Droplet vitrification	213	Nagel, personal communication, 2020
National Agrobiodiversity Center (NAAS), RDA, Suwon	South Korea	Garlic	• Droplet vitrification	1158	[85]
National Institute of Agrobiological Sciences (NIAS), Tsukuba	Japan	Mulberry	• Dormant bud freezing	1236	[80]
Tissue Culture and Cryopreservation Unit, NBPGR, Delhi	India	Mulberry	• Dormant bud freezing	329	[80]
USDA-ARS, Ford Collins and Corvallis	USA	Citrus	• Droplet vitrification	451	[86]
USDA-ARS, Ford Collins and Corvallis	USA	Apple	• Dormant bud freezing	2155	[87]

#### 4.1.1. Dormant Bud Cryopreservation

The number of crops or plants species that can be cryopreserved through dormant bud cryopreservation is limited since two requirements must be met: (i) the species produces buds that go into a dormant phase, usually induced in winter by a prolonged period of low temperature and/or photoperiod [88], before being prepared for cryostorage, and (ii) buds recovered from cryopreservation should respond to bud grafting.

A typical protocol for apple consists of (i) collecting dormant field material in mid-winter, (ii) air-dehydrating the twigs at −5 °C to 25–30% moisture content, (iii) subsequently applying slow freezing at 1 °C h^−1^ to −30 °C and holding this temperature for one hour, before (iv) the twigs are plunged into liquid nitrogen for storage [89]. After rewarming, buds are grafted onto a suitable rootstock. A comparative advantage of this protocol over the other cryopreservation protocols is that, during the whole procedure, no in vitro culture phase is involved; the material for conservation is transferred, after a proper treatment, from the field to the liquid nitrogen tank and, at the time of recovering, back from the tank to the field. This model saves much time [90] and resources and reduces the risks of contamination relative to classic shoot tip-based cryopreservation methods. To date, only apple, pear (*Pyrus* sp.) [91] and sour cherry (*Prunus cerasus*) [92] have been prepared for conservation with this method. An adapted dormant bud cryopreservation protocol where cryopreserved dormant buds are not grafted but directly planted out is developed for *Vaccinium* [87]. Alternatively, buds can be recovered in vitro as in the case of mulberry (*Morus* sp.) [93] and currants (*Ribes* sp.) [94].

#### 4.1.2. Droplet Vitrification

Droplet vitrification can be considered as a “generic” cryopreservation protocol for hydrated tissues, such as in vitro cultures [77,95], as opposed to dry tissues such as seeds and dormant buds. Because of its resulting high post-thaw regeneration rates, relative user-friendliness, and applicability to many plants species, it is now the widest applied protocol for cryopreserved germplasm collections. In short, 1 to 2 mm meristem tips are excised, precultured (or not) on a sucrose medium and treated with two highly concentrated liquids called loading solution (LS) and plant vitrification solution (PVS2 or PVS3) leading to a more concentrated (and thus vitrifiable) cell solution. The dehydrated meristem tips are transferred onto a small strip of aluminum foil and directly plunged in liquid nitrogen for storage. Rapid rewarming takes place in a third liquid, called recovery solution (RS) after which the meristems are transferred onto an in vitro medium for plant recovery. Since its first report in 2005 (See Box 2 [77]), this cryopreservation protocols has now been successfully applied to 111 plant species according the publications reported in Web of Science (Situation 1 July 2020) among which some very important staple foods are found, such as potato [96,97,98], taro [99], cassava and yam [82], sweet potato [100], and also ornamentals (*Pelargonium* spp. [101], *Nandina domestica* [102]), fruit trees (*Actinidia chinensis* (kiwi fruit)) [103], medicinal plants (*Byrsonima intermedia*) [104] and conifers (*Sequoia sempervirens* (redwood) [105]). For most of these crops/species, cryocollections are being established.

A more recent development in plant cryopreservation is the establishment of two cryo-plate vitrification methods: the vitrification cryo-plate (V Cryo-plate [106]) and dehydration cryo-plate (D Cryo-plate [81,107]). These methods follow the same principles as the droplet vitrification method; small volumes (droplets) cool down more rapidly to the temperature of liquid nitrogen compared to big volumes. The main difference is that, with these recent methods, meristems are enclosed in tiny drops of calcium alginate placed on the aluminum plate before being dehydrated and subsequently plunged in liquid nitrogen. Some advantages of the V Cryo-plate and D Cryo-plate method are (1) simple procedure to store plates in liquid nitrogen, (2) processing large numbers at a time and (3) secured high cooling and heating speed [108]. Post-cryopreservation regeneration rates of these methods are not significantly different from the droplet vitrification method [81], therefore, the choice of method to use is personal preference.

Routine cryopreservation of crop collections began only a few decades ago. Currently, about 18 genebanks have cryopreserved crop collections [13,67,81,98,109]. It is estimated that about 100,000 unique accessions of vegetatively propagated and recalcitrant seed crops potentially need long-term conservation through cryopreservation while currently only about 10,000 accessions are cryopreserved [13].

### 4.2. Advantages of a Cryopreserved Collection: Safety Backup for Clonally Propagated Crops

Cryopreservation is a cost- and labor-efficient conservation method that insures genetic stability over time [70], it may also be used for establishing a backup. For orthodox seeds such a safety backup facility already exists—the Svalbard Global Seed Vault (SGSV) in Norway. This facility is built by the Norwegian government and operated by the “Global Crop Diversity Trust” and NordGen, to store safety duplicates of national and international seed collections [110] to protect the diversity from irreversible loss due to natural and human-caused disasters. The SGSV consists of chambers maintained at −18 °C, dug into a mountain in the permafrost on Spitzbergen island at the arctic circle. Currently more than one million seed samples are conserved there by many national and international research institutes and genebanks.

Clonal crops can be duplicated in the field or in vitro but the ultimate backup may include a cryopreserved duplication of the conserved accessions. An additional advantage of the cryopreserved backup compared to the global seed vault is that the cryopreservation backup would serve for hundreds of years and does not need to be regenerated after a few decades of storage, depending on the species after 50 to 100 years, as is the case for seeds. Periodic regeneration of cryopreserved tissues is, however, necessary to evaluate successful conservation.

In 2017, a feasibility study concluded that a safety backup facility is currently required to accommodate 5000–10,000 accessions arising from ongoing cryopreservation activities [13] and that this facility should be expanded in a later phase to host all unique clonally propagated crop accessions. Such facility should operate according to the same policies and principles that govern the SGSV.

### 4.3. Problems Associated with Cryopreserved Collections

The range of crops represented in “cryobanks” is still rather restricted, and more than 90% of these accessions are composed of a few crops such as potatoes, cassava, apple, bananas and plantains, mulberry, garlic and strawberry. The main reasons why cryopreservation for the long-term conservation of vegetatively propagated crops is not applied more widely and on a larger scale are reviewed in detail by [13]. These challenges are linked to (i) protocol development: for many plant species efficient cryopreservation protocols are not available yet, (ii) problems with implementation of existing cryopreservation protocols such as genotype-specific responses and insufficient supply of healthy plant material, and (iii) challenges related to cryobanking capacities such as insufficient funding, lack of skilled personnel with knowledge on plant genetic resources [111] and lack of equipment/infrastructure.

## 5. Identification of Unique Accessions and Elimination of Duplicates

The elimination of duplicates in clonal collections has been an ongoing task since their establishment because (i) core collections need to be set up that contain the widest possible genetic diversity within the smallest number of accessions, and (ii) costs associated with maintaining clonal collections are high. CIP initially gathered more than 15,000 accessions of native potato cultivars from Latin America. Morphological characteristics and electrophoretic bands were used to identify duplicates and to reduce the collections to about 3500 accessions [17]. A reduction of 13% of the duplicates found in white clover resulted in a per accession saving of USD 500 regeneration cost in the field [112]. Additionally, other collections were screened for their redundancies. In eight Dutch apple collections, molecular fingerprinting revealed that 32% of the accessions were duplicates [113], a similar ratio to the duplicate proportion has been found in a barley seed collection [114]. In the US NPGS, about 12.5% of 1910 apple accessions were considered to be identical [115] comparable to the redundancy of a natural population of cacao [116]. Current next-generation sequencing technologies allow an increase in the number of studied alleles from about 10 SSR markers to several thousand SNPs and elucidate the relationship and ancestries between accessions and species resulting in a better identification of duplicates. The elimination of valuable genotypes has thus to be carefully balanced against the cost of the active preservation.

## 6. Costs Associated with the Different Conservation Methods

The aim, costs and safety considerations of a collection are important arguments in defining the necessary conservation approaches. To overcome the major drawbacks of field conservation, in vitro and especially cryopreservation are an option to secure clonal crop collections. The introduction of the genetic resources into in vitro conditions or cryopreservation requires well-equipped laboratories and trained personnel. It also requires the development of crop–specific growth media, the optimization of growth conditions, and the development of cryopreservation protocols. In 2000, the maintenance costs for one cassava accession at CIAT, Colombia, was estimated to be USD 7, USD 25, and USD 43 in the field, in vitro and in cryo conditions, respectively [14]. For garlic, the cost to maintain nine garlic plants (one accession) in the field and replant them annually is EUR 47, about 81% of this amount is due to labor costs. The introduction of one garlic accession into cryopreservation is about EUR 363, of which 52% is labor costs. The type of plant material has a strong impact on the cost of cryopreservation. When bulbils/inflorescences can be used, about EUR 318 is required whereas when cryopreservation is carried out with in vitro plants, the costs can increase to EUR 433 and up to EUR 557. The total depends on the number of in vitro subcultures required to achieve the appropriate number of plants for cryopreservation. Subsequent maintenance of such cryopreserved accessions, however, is rather low since this only demands regular filling up of the cryotanks with liquid nitrogen. Therefore, the break-even point when costs of field conservation are equal to that of cryopreservation is achieved after 8 to 13 years [21]. This estimate is very similar to the calculation made for bananas where the cumulative costs between in vitro and in cryo equate after 15 years. In general, the in vitro initiation of a banana accession at the ITC Leuven, Belgium, costs USD 861. The cost for cryopreserving such an accession is USD 1296. The costs accumulate differentially and are assumed in-perpetuity to reach USD 3686 and USD 1431, respectively. Nowadays, only few in vitro collections, such as the 1200 garlic accessions in Korea [85], about 150 mint accessions at IPK [67] and 150 citrus accessions in NPGS are completely preserved through cryopreservation [117]. However, it has to be considered that the material is only immediately available for distribution, characterization and evaluation when kept either in the field or in vitro.

## 7. Conclusions

The objective of a genebank is to conserve crop genetic resources. The advantage of a field genebank is that characterization and evaluation can be performed on mature plants at limited additional cost. Yet, the method has significant restrictions regarding its security, costs, and sustainability [14]. In this respect, in vitro conservation offers advantages over field conservation and is recognized as an invaluable complementary approach to secure the diversity of the collection. With the potential that tissue culture protocols can be developed for practically any plant species, the technique has been successfully applied over the past decades for short- to medium-term conservation of a wide range of crop species [118]. While offering the possibility of disease elimination and rapid clonal propagation of healthy plants, it is a suitable method for exchanging germplasm accommodating national and international phytosanitary requirements and regulations. However, the possible occurrence of genetic instability as a result of enhanced selection pressure under in vitro conditions compared to in vivo may be an obstacle to its use for long-term preservation of plant germplasm [49]. The chance that somatic mutations occur in tissues maintained at the ultra-low storage temperature of liquid nitrogen (−196 °C) is very slim since all metabolic processes are suspended [119]. In this regard, cryopreservation is the best option for unlimited and secure conservation of regenerative tissues in the long term. Nevertheless, the latter also requires adequate infrastructure, reliable electricity provision and well-trained staff.

The global COVID-19 pandemic has reminded us that particularly clonal ex situ collections in the field and in vitro that require continuous maintenance are vulnerable and insufficiently secured. “Essential” activities for maintenance of the germplasm in gene banks were temporarily interrupted for concerns out of human safety and health reasons, hence putting valuable genetic resources at risk of loss. While the initial costs of developing a crop- or species-specific cryopreservation protocol [120] and labor input for processing the samples in liquid nitrogen are high, once established, the maintenance of a cryopreserved collection requires very little input of resources including human intervention, making it probably the most cost-effective and secure option for long-term conservation.
